# Pilot Model for Community Based Oral Cancer Screening Program: Outcome from 4 Northeastern Provinces in Thailand

**DOI:** 10.3390/ijerph18179390

**Published:** 2021-09-06

**Authors:** Boworn Klongnoi, Vanvisa Sresumatchai, Siribang-on Piboonniyom Khovidhunkit, Pornpoj Fuangtharnthip, Rachatawan Leelarungsun, Binit Shrestha

**Affiliations:** 1 Department of Oral and Maxillofacial Surgery, Faculty of Dentistry, Mahidol University, 6 Yothi Street, Ratchathewi District, Bangkok 10400, Thailand; 2 Department of Biostatistics, Faculty of Public Health, Mahidol University, 420/1 Ratchawithi Road, Ratchathewi District, Bangkok 10400, Thailand; vanvisa.sre@mahidol.ac.th; 3 Department of Advanced General Dentistry, Faculty of Dentistry, Mahidol University, 6 Yothi Street, Ratchathewi District, Bangkok 10400, Thailand; siribangon.pib@mahidol.ac.th (S.-o.P.K.); pornpoj.fun@mahidol.ac.th (P.F.); rachatawan.lee@mahidol.ac.th (R.L.); 4 Maxillofacial Prosthetic Unit, Department of Prosthodontics, Faculty of Dentistry, Mahidol University, 6 Yothi Street, Ratchathewi District, Bangkok 10400, Thailand; binit.shr@mahidol.ac.th

**Keywords:** community screening, oral cancer, oral potentially malignant disorders, village health volunteers, cancer screening

## Abstract

Management of advanced-stage oral cancer adds a great burden to individuals and health care systems. Community-based oral cancer screening can be beneficial in early detection and treatment. In this study, a novel oral cancer screening program was conducted utilizing an existing network of health care personnel, facilities, and digital database management for efficient coverage of a large population. The screening program considered 392,396 individuals aged ≥40 from four northeastern provinces in Thailand. Three levels of screening were performed: S1 by village healthcare volunteers to identify risk groups, S2 by dental auxiliaries to visually identify abnormal oral lesions, and S3 by dentists for final diagnosis and management. A total of 349,318 individuals were interviewed for S1, and 192,688 were identified as a risk group. For S2, 88,201 individuals appeared, and 2969 were further referred. Out of 1779 individuals who appeared for S3, oral potentially malignant disorders (OPMDs) were identified in 544, non-OPMDs in 1047, doubtful lesions in 52, and no results in 136 individuals. Final treatment was carried out in 704 individuals that included biopsies of 504 lesions, exhibiting 25 cancerous lesions and 298 OPMDs. This study is so far one of the largest oral cancer screening programs conducted in Thailand and showed effective implementation of community-based oral cancer screening.

## 1. Introduction

**Figure 1:**
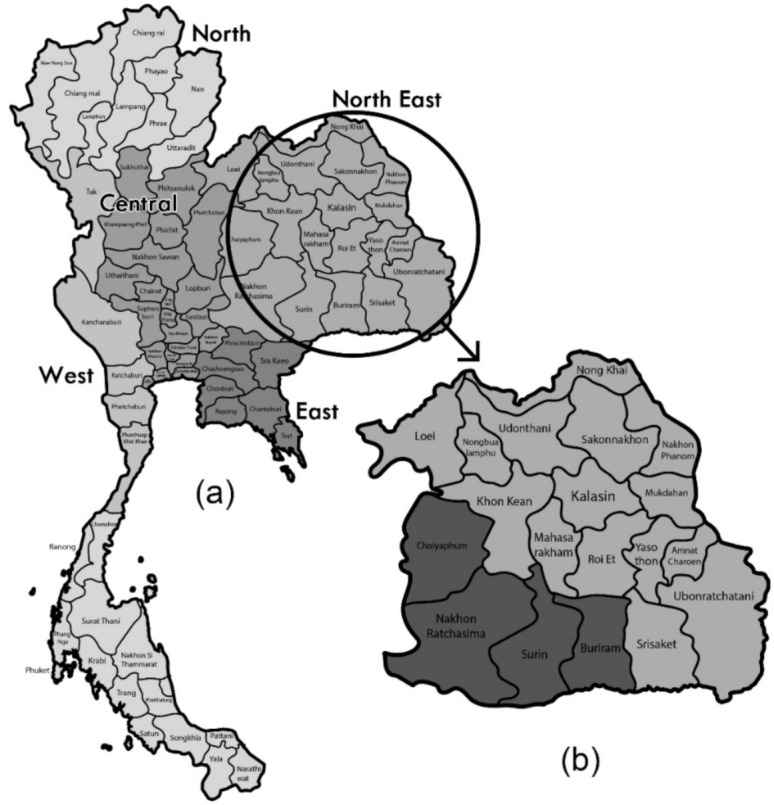
(**a**) Geographic map of Thailand; (**b**) northeastern provinces of Thailand with provinces considered for this study shaded dark.

Oral cancer is the sixth most prevalent cancer in the world [1], and accounted for an estimated 350,000 new cases and 177,000 deaths in 2018 [2]. Thailand is a country in Southeast Asia comprising 77 provinces that are arranged over five distinct geographical regions—Northeast, North, West, Central, and South (Figure 1). Oral cancer was reported as the eighth most leading form of cancer in Thai men and accounted for an ASR incidence of 4.6 and 3.2 cases per 100,000 males and females, respectively [3]. A 2018 Thai multicenter histopathological study reported squamous cell carcinoma as the most prevalent lesion (67%) [4]. It is estimated that by 2040 the incidence of lip and oral cavity cancer will increase by almost 65% from the current estimates [5].

Oral cancer is considered a lifestyle disease. Behaviors such as alcohol consumption and smoking have been primarily associated with increased risk of oral cancer. Exposure to human papillomavirus (HPV), syphilis, oro-dental factors, dietary deficiencies, chronic candidiasis, and viruses are also important predisposing factors [6]. Although globally declining in trend, the traditional habit of betel nut chewing is still prevalent in many parts of Thailand and has been known to contribute to the risk [7]. In the low socioeconomic population, knowledge and awareness of oral health may be limited, which may be further exasperated by the rural setting where timely diagnosis and treatment may be restricted due to lack of easily accessible health care service providers and facilities.

Early detection of oral cancer is important as diagnosis at late stages would significantly increase the treatment cost and associated morbidity and mortality rates. In several previous reports, more than half of the oral carcinomas were diagnosed at late stages (TNM Stages III and IV) [8,9,10]. A retrospective analysis of the Cancer Registry of Maharaj Nakorn Chiang Mai Hospital, Thailand, showed a higher predilection towards late diagnosis of oral squamous cell carcinoma, especially in patients aged ≥40. Only 3/8 of the confirmed diagnoses occurred at Stage I or II, whereas 5/8 occurred at Stage III or IV [11]. Similarly, a cohort study of 88 patients identified the male gender as a risk factor for late-stage diagnosis with strong associations with advanced stages and moderate–poor differentiation [9]. Late detection can also be attributed to the asymptomatic nature of the disease, especially during its early stages. Even when symptoms arise, patients may delay seeking medical consultation, which can further complicate the treatment [12]. From the population-based cancer registry of Khon Kaen Province, the five-year survival rate of oral cancer was reported as 64.9% for Stage I and 13% for Stage IV [13]. Therefore, initiatives for cancer prevention, especially early detection and intervention, are important to lessen cancer-related burdens on individuals and society.

Various forms of oral cancer screening and prevention have been described in the literature [14,15]. Screening of the general population for oral cancer is considered less effective due to its low incidence. Oral cancer screening programs that are designed for large populations mostly focus on primary prevention and on identifying individuals with high-risk behaviors in the general population. However, due to the cost involved, only a few nations have been able to implement it as a part of their general health screening program.

In Thailand, several studies have been conducted on oral cancer screening [16,17], but most of them have been based on small populations. Therefore, in this study, a novel community-based multi-level oral cancer screening program was carried out on a large population. The screening was designed for efficient utilization of the country′s existing network of heath care workers and facilities in conjunction with a digital patient registry. This pilot study focused on the screening of a randomized cluster of population from four provinces in Northeastern Thailand, and the results of the screening program have been reported.

## 2. Materials and Methods

This study was conducted from August 2019–June 2021. Population data for the study were obtained from an existing digital patient registry database provided by the Ministry of Public Health, Thailand (HosXP), and included individuals who were ≥40 years old and residing in Buriram, Chaiyaphum, Nakhorn-Ratchasima, and Surin provinces (Figure 1). Prior to the study, consultations and dialogues with the local administration were carried out at sub-district/district levels regarding the objectives of the screening program, and approvals were sought from the local community. Local village healthcare volunteers (VHVs) were assigned according to their corresponding villages. The first “S1” screening was performed by the VHVs to analyze individuals′ risk behaviors and was carried out at individuals’ homes. After attaining their consent, population demographics were attained along with an objective questionnaire-based interview to assess their risk behaviors. The risk factors were categorized into 8 parts as follows—current or past history of (a) smoking, (b) chewing tobacco or snuffing, (c) alcohol consumption, (d) betel quid consumption, (e) long durations of sun exposure while working, (f) denture wear for >1 year, (g) irritation of oral mucosa, and/or (h) history of head and neck cancer.

Individuals with ≥1 positive factor were deemed a risk group and referred for visual examination by trained dental auxiliaries at “S2” screening at sub-district level hospitals. These dental auxiliaries had received prior formal education on dental hygiene and were well capable of carrying out minor dental procedures. Prior to the study, they also received training by specialists in oral medicine. During S2 screening, they carried out thorough visual examination of the oral cavity with standardized instruments under light illumination for signs of abnormalities and the presence of red or white lesions using a standardized form provided by the study.

In the presence of suspicious or uncertain lesions, referrals were made to dentists at district-level hospitals for further investigation. At “S3” screening, dentists carried out the necessary intra- and extra-oral examinations, including palpation of lymph nodes and radiographic and histopathologic investigations, as deemed necessary. All relevant findings along with the treatment records were added to the digital registry.

**Figure 2:**
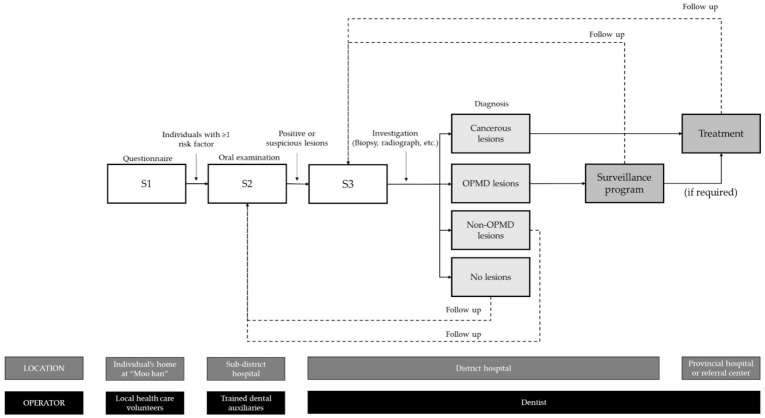
Screening methodology used in this study. OPMD = Oral potentially malignant disorder.

Oral potentially malignant disorder (OPMD) lesions were categorized as oral leukoplakia, erythroplakia, oral submucosal fibrosis, erythroleukoplakia, and verrucous hyperplasia [18], whereas non-OPMD lesions included fibrous hyperplasia, irritating fibroma, pyogenic granuloma, etc. For both OPMD and non-OPMD lesions, primary treatment included medication, laser therapy, removal of irritation, and minor surgeries. Individuals with cancerous lesions were then referred to provincial hospitals for necessary management. Individuals with OPMDs were followed up at the S3 level, where they were recalled every 6 months for continual examination. Individuals with non-OPMD lesions, no lesions, or without a confirmatory diagnosis were referred to the S2 level for regular follow-up (Figure 2). To aid in patient database registry and management, a customized software module designed by the oral cancer screening program was added to the existing HosXP program. The program allowed data entry and retrieval regarding the risk factors recorded from S1 and S2 levels, characteristics of the lesions found in S2 and S3 levels, and the treatment received by each patient at the S3 level.

## 3. Results

**Figure 3:**
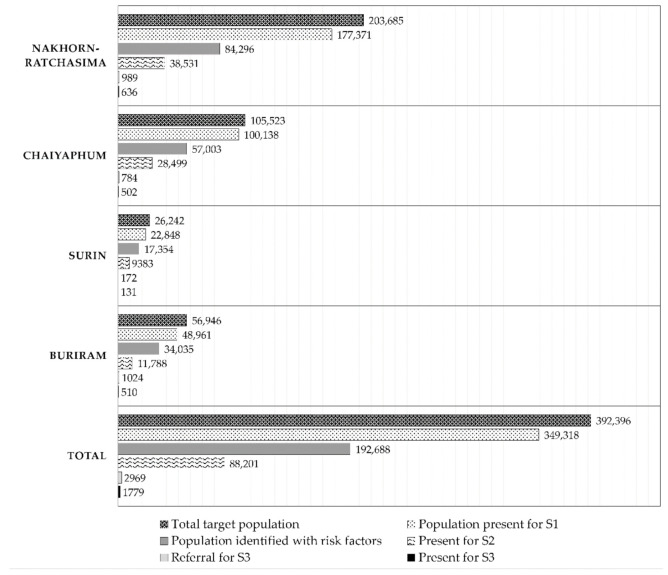
Results of screenings 1–3 in this study.

The study took into consideration a target population of 392,396 individuals from a total of 81 sub-districts from 13 districts of 4 provinces. A total of 26,921 VHVs were mobilized for the study with an average target population, with a healthcare volunteer ratio of 15:1 (Table 1). Out of the total target population, 349,318 enrolled in the study for S1 screening (Figure 3).

**Figure 4:**
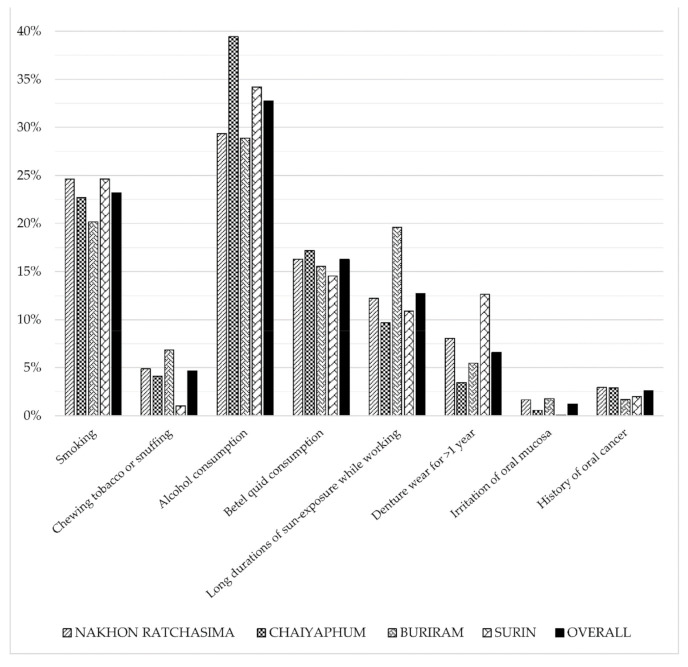
Prevalence of risk factors in individuals present at S1.

From them, 192,688 individuals were identified as the risk group and comprised approximately half of the individuals screened for S1. The results of the analysis of the risk factors prevalent among the individuals present in S1 screening were as shown in Figure 4. The most prevalent risk factor in this population was alcohol consumption (33.8%), followed by smoking (23.2%), betel quid consumption (16.3%), long duration of sun exposure (12.7%), denture wear >1 year (6.6%), chewing tobacco or snuffing (4.7%), history of oral cancer (2.6%), and irritation of oral mucosa (1.2%). All four provinces had smoking prevalent in >20% of the study population, except Nakhon Ratchasima and Surin, which exhibited a prevalence rate of 24.6%. Alcohol consumption was most prevalent in Chaiyapum at 39.4%, followed by Surin at 34.2%. Betel quid consumption was evenly present in all provinces with a prevalence rate of approximately 15%, except Chaiyaphum at 17.2%. On the other hand, long duration of exposure to the sun was the highest in the study population from Buriram at approximately 20%. The overall prevalence of chewing tobacco or snuffing was 4.7%, whereas the prevalence of denture wear, irritation of oral mucosa, and history of oral cancer was 6.6%, 1.2%, and 2.6%, respectively.

In S2-level screening at local sub-district health centers, 88,201 individuals participated with a dropout rate of 45.8% from S1. From this population, a total of 2969 individuals with abnormal or doubtful lesions were identified and further referred for S3-level screening (Figure 3). For S3 screening, 1779 individuals participated with a dropout rate of 40%. Out of them, 544 were diagnosed with OPMDs, 1047 with non-OPMDs, and 52 with doubtful lesions, and 136 had no diagnosis (Table 2). From them, 704 were treated, which included 504 biopsies that exhibited 25 cancerous lesions, 298 OPMDs, and 181 non-OPMDs, as shown in Table 3. The biopsy results showed confirmatory diagnosis of squamous cell carcinoma in 14 (56%) lesions, which were obtained from 12 individuals (mean age of 66.8 years, age range 50–95), and verrucous carcinoma in 6 (24%) lesions. The histopathological reports of the cancerous lesions along with patient demographics are summarized in Table 4. The majority of the OPMDs comprised mild epithelial dysplasia (45%), oral lichen/lichenoid planus (21%), and moderate epithelial dysplasia (13.5%) as shown in Table S1, whereas non-OPMDs comprised fibroepithelial hyperplasia (42%), squamous papilloma (10.5%), and giant cell fibroma (8%) as shown in Table S2.

## 4. Discussion

The northeastern region of Thailand comprises 20 provinces with a total population of 22,240,574 (2018). This pilot study focused on four neighboring provinces and included a total of 13 districts with a cumulative population of 931,084 individuals. The target population aged ≥40 accounted for 42% (392,824) of this population. The WHO (2011) reported that the overall prevalence rate of smokers among the general Thai adult population was 24%, with a higher prevalence in rural areas and among males. On the contrary, the use of smokeless tobacco was more prevalent in females. Considering the regional data, the overall prevalence of tobacco use in the northeastern region was second only to the southern region [19].

The association between oral cancer and tobacco smoking, alcohol consumption, and betel quid chewing has been well established [20]. Betel quid is traditionally prepared by wrapping areca nut with fresh or fermented betel leaf with lime, tobacco, or other regional additives [21]. Although primarily consumed for its psychostimulant properties, reca alkaloids (arecoline and arecaidine) present in areca nut can be a major source of toxicity [7]. Betel nut consumption and smoking was reported to increase the risk of developing oral premalignant lesion by eight- to nine-fold than those who refrained [16]. Most of the individuals in the northeastern region of Thailand rely on agriculture for their livelihood and are exposed to prolonged durations of sunlight, increasing their overall risk of cancer [22]. Alcohol consumption, smoking, betel quid consumption, and long durations of sun exposure were the most prevalent risk factors among the individuals who appeared for the initial S1 screening in our study. These data also agreed with a previous report by the National Statistics Office, which reported a prevalence rate of 21.1% for smoking and 32.8% for alcohol consumption in the northeastern region [23]. Similarly, a survey on the use of smokeless tobacco in Thailand reported that the prevalence rate of betel quid consumption was the highest in rural communities and in the age group >65 years at 8.2% compared to other age groups. Comparatively, a higher prevalence of betel quid consumption (16.3%) and the use of chewing tobacco or snuffing (4.7%) was observed in our results, which could have been due to the region and age group-specific nature of the study. Furthermore, both past and current users or experiences were grouped as one in our analysis of the risk factors. In general, the prevalence of smokeless tobacco consumption in Thailand remains low (3.2%) compared to other countries such as Bangladesh (27.2%) and India (25.9%) [24].

The screening of a large group of individuals for early detection of oral cancer can be challenging due to the required resources, capable personnel, and facilities. Opportunistic screening of oral cancer can be performed when individuals visit dental professionals. This method of screening is considered cost effective to identify potential lesions [25]; however, in developing countries the number of individuals who regularly visit dental clinics is low, and it is still questionable whether these individuals represent the general population. Self-examination of the oral mucosa by the individuals themselves may prove difficult, as they may not be able to differentiate between normal and abnormal mucosa without appropriate education or visual media [26]. Therefore, to improve the overall effectiveness of this study, we first considered identifying high-risk individuals from the general population and then visually screening them for abnormalities.

Several large-population-based oral cancer screening programs have been conducted with various levels of success. In a study carried out in Gujrat, India, 5214 trained village healthcare workers screened 2,610,423 individuals and referred 3309 individuals to tertiary care centers, among which 1890 (57.1%) were diagnosed positive for malignancy [27]. Similarly, in a study conducted in Kannur, Kerala, 1,061,088 individuals were surveyed by 6325 volunteers who identified 3226 patients, out of which 2507 attended subsequent screenings, and 13 were identified with oral cancer and 174 with precancerous lesions [28]. A study by Chuang et al. followed up with individuals with habits of smoking and betel quid chewing in Taiwan. A questionnaire-based interview was followed up by visual examination by trained dentists. Out of 2,334,299 individuals who took part in the study, 16,494 were identified with suspicious lesions, among which 11,051 were identified with premalignant lesions and 4110 were identified with oral cancer. Subsequent evaluations during follow-up led to the identification of an additional 3923 lesions [29]. Besides initial screening, subsequent follow-ups of individuals, especially those with potentially pre-malignant lesions, play a pivotal role in the success of oral cancer studies.

Thailand has an existing extensive network of 800,000 (2012) [30] active VHVs, who are overseen by the Department of Health Service Support to provide basic health-related information at the local grassroots level. Many of them can speak local dialects and can encourage villagers to seek medical consultation through discussion and informal conversations. One such achievement attained by the VHVs in this study was a high compliance from the target population into S1 (89%). Unfortunately, at each level of screening dropouts were observed: S1 to S2—54% and S2 to S3—40%. This could have been due to the rural backdrop, where individuals cannot spare their daily wage or traveling time to visit a distant healthcare facility for health issues that may not be of importance to them. At the final S3 screening, 704 individuals were treated and 745 were placed under observation. Biopsy results revealed 25 cancerous lesions in 21 individuals and 298 OPMD lesions. Although the cancer lesions were mostly prevalent in the fifth to eighth decade of life, one third occurred in the seventh decade, which was in conjunction with a previous multicenter histopathological study of oral malignant lesions [4].

This has been so far the largest documented observation of malignant and pre-malignant lesions in Thailand. In a similar community-based oral cancer study carried out in five districts of Roi-et province in Thailand, 57,763 adults were sent self-screening forms and questionnaires, out of which 3428 responded positively and were subsequently examined by dental nurses. Only 201 out of 407 who were referred appeared for their appointment with dentists. Ninety-nine patients were then referred to an oral and maxillofacial surgeon at a provincial hospital. Although 88 appeared, only 10 individuals accepted biopsy, and the results showed 1 cancerous lesion and 6 PMDs [17]. Compared to the Roi-et study, this study utilized a three-tier screening process, which improved the workflow by reducing redundant steps both for the patient and treatment team. It allowed the hospitals and dentists to focus on diagnosis and treatment rather than screening. Treatments such as medication, biopsy, laser therapy, removal of irritation, and minor surgery were performed at district hospitals with visiting treatment teams from central or regional hospitals, which also created opportunities for learning, collaboration, and knowledge sharing among the teams. Only patients with advanced cancerous lesions were referred for proper treatment at the central or regional hospital.

Cost utility analysis compares the cost of an intervention to its effectiveness measured in natural health units. For an intervention to be cost effective, the cost of the intervention per disability-adjusted life year should be less than three times the country′s annual gross domestic product (GDP) per capita [31]. In context with Thailand, a 2013 report established the ceiling threshold of cost effectiveness at THB 160,000 for each quality-adjusted life year (QALY) gained [32]. Similarly, Kumdee et al. calculated the costs of diagnosis, treatment, and follow-up of oral cancer patients at Roi et Hospital, Thailand, and attained an incremental cost-effective ratio (ICER) of THB 311,030 per QALY, which only had a 30% probability of being cost effective compared to no-screening [33]. Our preliminary findings on the cost effectiveness, attained in terms of QALYs calculated by multiplying the life expectancy by utility value, attained an ICER of THB 135,314.30, which was 0.53 times the current GDP of Thailand (USD 7804.74, 1 USD = THB 32.84). These results are indicative of a highly effective intervention, and a detailed report is in progress.

For future implementation, it is suggested to adjust the criteria for S1 screening for efficient identification of high-risk individuals from the others and to lessen the workload on S2-level screening. One method of attaining effective recruitment would be to segregate the risk factors into major and minor risk factors, and to only recruit individuals with risk factors that have the highest association with oral cancer and OPMDs, such as alcohol consumption, tobacco smoking, use of smokeless tobacco, and betel quid chewing. Exposure to multiple risk factors can also be considered during recruitment into S2, as synergistic effects among risk factors were observed in our previous study [34]. These changes in recruitment could further improve the study′s cost effectiveness and reduce potential dropouts.

The digital patient registry also simplified tracking individuals’ progress from the S1 to S3 level. Although the accessibility of the system was greatly improved via internet-enabled terminals, it was also prone to human error. At S3-level screening, 136 individuals were labeled under “no diagnosis” due to improper or lack of data entry. To improve the effectiveness of the study, data entry should be strictly implemented. Even though oral cancer screening by visual examination is considered to have high specificity and sensitivity [35], some potentially harmful lesions could have been missed at the S2 level. Moreover, the study identified 544 individuals with OPMDs, which showed the necessity for a robust follow-up system for early detection of malignant transformations. In order to reduce dropouts and to improve the compliance of patients under the surveillance program and follow-ups, an automated module is currently under development that would send digital text-based reminders (SMS) to the patients and treatment team regarding their appointments [36]. However, its results, comparisons to conventional telephone-based reminders, and implications are yet to be determined.

## 5. Conclusions

Our oral screening program aimed for complete management, where individuals with cancerous or premalignant lesions were screened, diagnosed, treated, and followed up with using standard protocols. The results of this pilot model have created one of the largest oral cancer databases in Thailand and showed that a community-based screening model utilizing VHVs, dental auxiliaries, and dentists with a digital database management could be effective in oral cancer screening of a large population. The model also has the potential to be applied for the screening of other forms of cancer. However, it may be best suited for countries with an existing network of health care workers and facilities.

## Figures and Tables

**Table 1:** Demographics of each district considered for the study.

Province	District	Total Population	Target Population Aged ≥ 40	No. of VHVs	Target Population: VHVs
Nakhorn-Ratchasima	Non Sung	85,091	48,470	2565	19
	Prathai	71,433	29,555	1703	17
	Kaam Thale So	25,902	8491	774	11
	Pra Thong Kham	36,688	14,175	1041	14
	Non Thai	54,699	26,396	1803	15
	Mueang Nakhon Ratchasima	232,838	76,598	8107	9
Buriram	Lahan Sai	19,463	24,714	1319	19
	Krasang	101,637	32,232	1663	19
Surin	Si Narong	46,803	13,589	832	16
	Khwao Sinarin	34,361	12,653	748	17
Chaiyaphum	Phu Khiao	108,678	49,399	2935	17
	Kaeng Khro	72,820	35,691	1848	19
	Bamnet Narong	40,671	20,433	1583	13
Total		931,084	392,396	26,921	15 (mean ratio)

VHV = Village healthcare volunteers.

**Table 2:** Results of individuals diagnosed and treated at the S3 level.

Province	Diagnosis					Treatment	
	OPMDs	Non-OPMDs	Doubtful	No Diagnosis	Observed	Treated ^1^	Biopsy in Treated Group
Nakhorn-Ratchasima	156	438	15	27	316	199	180
Buriram	213	238	13	46	183	275	166
Surin	53	49	1	28	45	55	48
Chaiyaphum	122	322	23	35	201	175	110
Total	544	1047	52	136	745	704	504

^1^ Treatment included medication, biopsy, laser therapy, removal of irritation, or minor surgery.

**Table 3:** Results of biopsies (number of lesions).

Province	Cancerous Lesions	OPMDs	Non-OPMDs	Total
Nakhorn-Ratchasima	12	87	81	180
Buriram	7	100	59	166
Surin	3	34	11	48
Chaiyaphum	3	77	30	110
Total	25	298	181	504

**Table 4:** Histopathological report of cancerous lesions with patient demographics.

Lesion Number	Gender	Age	Diagnosis	Type	Location
1	M	64	SCC	Micro invasive	Buccal mucosa
2	F	70	VCC versus verrucous hyperplasia		Buccal mucosa
3	F	73	Clear cell odontogenic carcinoma		Palatal gingiva
4	F	54	Mucoepidermoid carcinoma	Low grade	Retromolar area
5	F	50	Basal cell carcinoma		Nose
6	F	80	Adenoid cystic carcinoma		Lower lip
7	M	55	Lymphoma		Palatal mucosa
8	F	86	VCC		Retromolar area
9	M	67	SCC	WD	Lower lip
10			SCC	NS	Right vermillion border
11	M	65	SCC	Micro invasive	Ventral border of tongue, floor of mouth
12	M	56	SCC	WD	Retromolar area
13	M	59	SCC	NS	Lateral tongue
14	M	71	VCC		Buccal mucosa
15	F	73	SCC	WD	Lower lip
16	F	95	SCC	NS	Lower lip
17	M	86	SCC	Micro invasive	Lower lip
18	M	89	SCC	WD	Soft palate, uvula
19	F	79	VCC		Upper lip
20			VCC		Upper gums
21			VCC		Lower lip
22	M	69	SCC	NS	Lateral Tongue
23	F	76	SCC	NS	Palate
24	F	76	SCC	WD	Lower lip
25			SCC	WD	Upper lip

SCC = squamous cell carcinoma, VCC = verrucous cell carcinoma, WD = well-differentiated, NS = not specified.

## Data Availability

Data can be made available on request by email.

## Supplementary Materials

The following are available online at https://www.mdpi.com/article/10.3390/ijerph18179390/s1, Table S1: Histopathological report of OMPDs and their frequency, Table S2: Histopathological report of non-OPMDs and their frequency.
