# Supplementary material for: Pilot Model for Community Based Oral Cancer Screening Program: Outcome from 4 Northeastern Provinces in Thailand
Source: Int J Environ Res Public Health. 2021 Sep 6;18(17):9390. doi: 10.3390/ijerph18179390 (PMC8430625; doi:10.3390/ijerph18179390)
Supplement: Supplementary file 1 [file ijerph-18-09390-s001.zip › ijerph-1357825-supplementary.pdf]

International Journal of
Environmental Research and Public Health

MDPI

SUPPLEMENTARY MATERIALS

Table S1. Histopathological report of OPMDs and their frequency

OPMDs	No. of lesions
Mild epithelial dysplasia	134
Oral Lichen/Lichenoid planus	62
Moderate epithelial dysplasia	40
Severe epithelial dysplasia	18
Hyperkeratosis	11
Acanthosis + Hyperkeratosis	6
Lupus erythematosus	6
Acanthotic epithelium	4
Hyperplasia	4
Verrucous hyperplasia	4
Actinic cheilitis	3
Hyperplastic candidiasis	3
Submucous fibrosis	2
Hyperplastic and hyperkeratinized	1
TOTAL	298

Table S2. Histopathological report of non-OPMDs and their frequency

Non-OPMDs	No. of lesions
Fibroepithelial hyperplasia	76
Squamous papilloma	19
Giant cell fibroma	14
Pyogenic granuloma	10
Inflamed tissues	10
Candidiasis	7
Inadequate specimen	3
Verruca vulgaris	3
Vascular malfarmation	2
Ulcer	2
Pleomorphic adenoma	2
Venous malformation	2
Vascular ieiomyoma	2
Traumatic neuroma	2
Vascular leiomyoma	1
Verruciform xanthoma	1
Oral melanotic macule	1
Epidermoid cyst	1
Lymphoepithelial cyst	1
Lingual tonsil hypertrophy	1

Granulation tissue	1
Radicular cyst	1
Schwannoma	1
Fibrous tissue and epithelium	1
Candida cheilitis	1
Traumatic ulcerative granuloma with stromal eosinophilia	1
Angiokeratoma	1
Intramucosal nevus	1
Epulis fissuratum	1
Intramuscular lipoma	1
Mucositis	1
Mucous membrane pemphigoid	1
Lipofibroma	1
Peripheral ameloblastic fibro-dentinoma	1
Cyst	1
Caliber-persistent artery	1
Neurofibroma	1
Oral mucosa	1
Mucocele	1
Superficial mucocele	1
Connective tissue	1
TOTAL	181
